# Indents

**Published:** 1908-11-15

**Authors:** 


### INDENTS.

☞ **Briet Articles of Technical or Scientific Value will receive notice and comment. Contributions invited.**

**Serum Treatment.** In the course of a letter in the *British Medical Journal* of August 15, dealing with the "Therapeutic Uses of Normal Serum," Dr. D. Montgomerie Paton. L. R. C. P., and S. Edin., of Melbourne, points out that in October, 1907, he reported to the Odontological Society of Melbourne over 300 cases of the local use of anti-diphtheritic serum in traumatic and septic dental conditions. The cases dated from 1901, since which year he has had a further batch of satisfactory results. The cases were reported in the *Australian Journal of Dentistry* for February, 1908.—*American Journal.*

**Mixing Investment for Inlays.** I think the investment and the mixing of the investment is very important; and I know that Dr. Taggart makes the claim that as nearly as possible, exact methods should be used in managing this investment, to which end he has designed a double-ended ladle, one end of which will contain the proper quantity of investment and the other end a proper quantity of water, and you will find that if you mix Taggart's investment in that proportion, you will have a very stiff mixture. Those of us who handle cement know oxyphosphate mixed thin is a cement; mixed thick it is a filling material. In other words, if you want strength, durability and density, you must put more material with your liquid. And so it must be with this investment material, and Dr. Taggart puts his investment over the fire and finishes the whole work as quickly as possible. Not only that, but in his flask ring he has a hole drilled and after the ring is filled he turns it upside down and presses against the investment material, so that the water oozes out and the investment is made more dense.—R. OTTOLENGUI, *Items of Interest.*

**Danger in Absorbent Cotton.** Absorbent Cotton is prepared in factories on a large scale. Raw cotton is carded, freed from grease by washing with soda, bleached with a hypochlorite, and finally washed with a dilute solution of sulphuric acid. It is then dried, made into packages and then "sterilized." After each preliminary operation it is washed with water, and for this purpose manufacturers use any water that comes handy, and in this way any number of microbes may be introduced into it. The insufficiency of the sterilizing is shown by the startling results of M. Nonnette's investigation of commercial brands marked "aseptic", and sterilized at 150 degrees centigrade. Cultures were made with every precaution of modern asepsis from thirty packages at random. In every instance flourishing colonies of mould, yeast and microbes of various kinds were obtained. Bacillus subtillis, bacillus coli, staphylococci, and streptococci appeared in all the cultures, and two of them yielded typhoid bacilli. These results prove that it is unsafe to apply commercial absorbent cotton to wounds, or in edema, erythema and other inflamed conditions of the skin. *Scientific American*, July 27, 1908, page 457.

**Sticky Wax.** The refuse beeswax of the laboratory can be utilized in making hard or sticky wax. First remove all paraffin, modeling compound, etc., and place the wax in a vessel with plenty of water and heat it until it boils. Continue the boiling for a few minutes, then set it aside to cool. When quite cold remove the cake of wax which will form over the water. The heavier impurities, plaster, etc., will have settled to the bottom of the vessel, the lighter dirt will be on the bottom of the cake and may be removed with a knife. Take of common resin about the weight of the wax, place it in a suitable vessel, which should be large enough to contain ten times the amount, and heat it until it melts. While it is still kept hot, add the wax in small portions, until by testing it, letting a drop fall into cold water, sufficient has been added. If intended for summer use less wax is needed than for use in winter. As a rule, when thoroughly cold it should be brittle. When the wax is added, it usually boils up, and if the vessel is not amply large it will boil over. The melting point of resin is much above that of beeswax. It may be made into rods about the size of a lead pencil while plastic, or run into plaster of paris molds kept thoroughly wet with cold water.—*Brief*

**Crown Helps.** After setting a crown of any kind, ofttimes pain follows, contrary to all care exercised in preparation, due to two distinct causes.

First, the contraction of cement, more especially the rapid setting, and the cases where the pulp is alive and dentin exposed. In those cases I find 25 per cent solution of silver nitrate treatment very good. Caution must be given to thoroughly dry the tooth before a gold crown is set to avoid discoloration.

Second, an abrasion or laceration of the soft tissues around the gingival border when preparing for crown, allowing an excess of acid from cement to penetrate the contused surface of the nerve terminals, causing pain. This pain can be controlled by taking saturated solution of bicarbonate of soda and immediately following with wine of opium over the gum. Cases of pericemental tenderness, usually following crowning of teeth, can be promptly relieved with Dr. Buckley's liniment, applied locally over affected gum area. The formula is:

| | |
|---|---|
| R Menthol, | grs. XX |
| Chloroform, | dr II |
| 10% Tinc. Aconite, | dr VI |

—Dr. W. G. Hamm, in *Summary.*

**Solidified Formaldehyde, Combined with Ceylon Oil of Cinnamon in a Sodium Glycerate Base, for Dental Purposes.**

Dr. David Genese, of Baltimore, Md., recommends this combination as an antiseptic dressing in pulp and pulp-canal treatment. Its effect is immediate and is continued. It forms a paste easily handled and can readily be carried where it is needed without risk of spreading to where it is not desired. On a fine, smooth broach wind a little cotton loosely, gauging the amount so that it will readily pass into the root canal; take up a little of the paste on the end of this and pass it into the canal; then by a reverse twist remove the broach, leaving the cotton in place. To seal it on another broach or probe lightly, secure sufficient cotton to make a secure seal; melt a little paraffin on a spatula, and dip the cotton into it, making it take up as much as it will, then quickly pass it through a flame and immediately carry it to position and press it to place with a blunt instrument. Passing the cotton through a flame thoroughly sterilizes it and burns off any loose outstanding fibers. The paraffin saturated cotton makes a clean, moisture-tight seal, non-absorbent, requires no pressure for its insertion, and is readily removed.—*Brief.*

**Thorough Mastication May be Overdone.**

Now and again thorough mastication has been urged as a means of getting the most from the food taken, on two grounds. First, it is better prepared for digestion and less is needed; second, less vital force is expended within the human machine, and, therefore, more is available for outside useful work. While this may be true on general principles, it is so to a limited extent only. Regarding this Metchnikoff says: "Certainly the habit of eating quickly favors the multiplication of microbes around the lumps of food which have been swallowed without sufficient mastication. It is quite harmful, however, to chew the food too long, and to swallow it only after it has been kept in the mouth for a considerable time. Too complete a use of the food material causes want of tone in the intestinal wall, from which as much harm may come as from imperfect mastication. Comparative physiology supplies us with arguments against too prolonged mastication. Ruminants, which carry it out to the fullest extent, are notable for extreme intestinal putrefaction and for the short duration of their lives. On the other hand, birds and reptiles, which have a very poor mechanism for breaking up food, enjoy much longer lives.—METCHNIKOFF, *The Prolongation of Life, English Translation*, page 159.—*Brief*.

**Erosion and Sensitiveness.** After the teeth are cleaned and the gums and mucous membranes massaged and the mouth made as nearly sterile as may be, the dam is placed and crowded as far up as possible to expose the entire eroded surface. The affected teeth are then freely bathed in hydrogen dioxid and the chemical dried into the tooth with a warm instrument. This is done to make use of both the cleansing and mechanical emptying properties of hydrogen dioxid, thus opening up and disinfecting the enlarged orifices of the tubili. After the agent is thoroughly dried, chloroform is freely used to wash away any fats that may be present and the tooth wiped dry. Then a stream of warm air is turned on till the tooth is dry. The tubuli now being as free from foreign substances as I can get them, 40 percent formaldehyde solution is applied plentifully and dried into the tooth as thoroughly as can be done with hot instruments and hot air, a bit of paraffin placed on the affected spot and melted there with the hot instrument and the heat carried as high as the patient can stand, the object being to get the paraffin as deeply into the tubuli as possible. Treatment repeated six or eight times two or three days apart and the case dismissed with instructions to return at once should sensitiveness again develop.

I know of nothing better thus far, but would be glad to hear from others as to their methods, successes and failures. —LUCIEN H. ARNOLD, in *Digest*.

**An Accurate and Safe Way of Applying Silver Salts.** Secure three sizes of smooth, flexible canal-dressing instruments of platinum, German silver, or the so-called platinoid material. Secure a small wide-mouthed bottle, and place in the same the granular silver salts. Heat your instrument slightly and plunge it into the bottle, taking up a crystal of any size that you may wish to use. Your crystal, which is now only slightly attached, is passed back and forth in a flame until it is thoroughly fused on the very tip of your instrument. Having thus been fused to the end of the instrument, the salt can in full strength be safely and accurately applied to either hard or soft tissues.

Silver nitrate is preferred by the clinician if discoloration be not objectionable.

In applying salts by this method it must be kept in mind that the salts are used full strength; the tissues must therefore be protected by the use of napkins and the saliva pump. If the spot to which the salt is applied is not moist enough to gradually dissolve the fused point, it is moistened with a little moist cotton. By having flexible instruments of three different sizes, the point of fused salt can be applied in varying sizes, which allows greater accuracy. The flexible instruments can be bent to any angle desired.—A. J. FLANAGAN, in *Cosmos*.

**Orthodontia Specialists.** The more we study malocclusion, the more we see the need of the specialist. Someone said in the discussion, "Let the dentist treat the simple cases." Gentlemen, there are no simple cases in orthodontia. Even the diagnosis of cases of malocclusion is not simple. It is true that rules for diagnosis were discussed and determined upon long before Dr. Angle brought out his great classification, but with what result? We find classes varying from six to twenty, based upon the position of almost every tooth in the mouth, except the one that is most stable, and therefore the correct one on which to base a diagnosis. Let us all give due honor to Dr. Angle, who has recognized this fact and has so reduced these classes to three; and, gentlemen, the sooner the importance of this fact is appreciated by the general practitioner, the better it will be for the patients under his treatment.

With regard to extraction, of course you all know that I do not believe in extraction. As far as its relations to charity work is concerned, I say when we give charity we want to do the very best we can for our patients, and if we extract the teeth we are usually doing the very worst thing we can do for them. Thus we create a new deformity in trying to correct the original one, and unfortunately the deformity created is worse than the one apparently corrected. —Dr. Strang, in August *Cosmos*.

**Separating Teeth.** A piece of floss silk is doubled and passed between the teeth and between the loop made by the doubled silk a piece of ordinary cotton twine that has been previously sterilized is passed into the loop; this is drawn through the loop and we have a loop of the twine and a loop of the silk held together as two links of a chain; the silk is now drawn back through the teeth, pulling the cotton twine with it. When the loop of twine appears through the teeth, one end of the silk is dropped and the silk is pulled out of the twine and thrown away. The silk is only used to facilitate the drawing of the double cotton twine between the teeth. If there is room to get the twine in without using the silk, it is of course not necessary to use it. The twine is now between the teeth with the loop toward the labial and the open ends toward the lingual surfaces of the teeth. One end of the twine is now grasped between the fingers, brought over the incisal surfaces of the teeth and slipped in between the loop, the other end is taken and the two ends united into a knot; as the knot is drawn tight the loop and the knot working toward each other cause the teeth to spread apart. A number of knots may be made as tightly as may seem advisable and the patient dismissed until the appointment for the filling. This method when learned is very easy for both patient and operator and causes as much separation as one may desire, for the thickness of the twine and the number of knots made can be suited to the case. When properly inserted it is very rare to have one of these separations come out before the operation.—J. W. CONZETT, in *Digest*.

**Electricity as an Anesthetic.** Before a gathering of homeopathic physicians in Flower Hospital, New York, Dr. William H. King, dean of the Homeopathic college, subjected a dog to an electric current to demonstrate that electricity can be used successfully in place of ether or chloroform as an anesthetic.

Dr. King got a black-and-tan terrior in good health, shaved the hair off the top of his head and his back near the tail above the lumbar region. One electrode was placed on the head and the other on the back and an intermittent current of a little more than six volts was turned on. The current was from the regular street supply, modified in the specially constructed apparatus used. In forty-five seconds the dog was unconscious. He could be handled without the least danger of awakening him. No operation was performed.

The somnolence continued only so long as the electrodes were kept in place. When they were removed the dog almost instantly regained consciousness, was as lively as before the current was turned on and a close examination could not discover the least ill effects.

The intermittency of the current produces the somnolence which makes it possible to perform operations without pain to the patient and no after-effects. It is the after-effects which make many persons hesitate or refuse to take ether or chloroform. A battery could be used in the place of the street current. The use of this current in surgical operations will mean a great advance in surgery. Many of the disagreeable features now attending it will be removed, and the patient will be in a better condition to be operated on."
—*Chicago Daily News.*

**Occlusion.** We are in the habit of expecting the orthodontist and the prosthodontist to to be well-versed in the science of occlusion, but the operative dentist in the past has thought that occlusion was of little importance to him. Not so; it is of the utmost importance to the permanence of his work, more than that, it is of importance from the standpoint of comfort, to the patient, and ability to properly use his dental organs. We frequently find cases in which there are cavities between every tooth in the upper jaw and perhaps many between the bicuspids and molars of the lower jaw. Now if in each of those cavities we fail to reproduce the normal contour by only .5 millimeter, that will make a full millimeter between each tooth, and when we multiply that by ten to sixteen teeth, you can begin to understand the havoc made in the relation of the teeth one to the other occlusally, and I assure you that the picture is not overdrawn, and if you will carefully examine the cases that come into your office with this in mind you will bear me out in the assertion that the *most important* thing in the filling of teeth is the reproduction of the proper contact and interproximal spacer I consider it good practice to remove a filling that is perfect in every other way if it is not made so that the tooth is able to take its proper place in the arch and perform its proper function; and it is not able to do that if its occlusal surface has lost from two to six millimeters of masticatory surface to say nothing of the havoc being wrought at the gingival margin. More than that, I consider it good practice to cut into perfectly sound teeth and build out a normal contact in those cases where the contact points have been worn down into a broad facet and food is able to crowd in between the teeth and into the interproximal space to the great discomfort of the patient.—J. W. CONZETT, in *Digest.*

**German Dental Decision.** According to Consul General Richard Guenther, the district court at Frankfort recently rendered the following interesting decisions in a suit between American dentists practicing in that German city.

A firm composed of two leading American dentists here about eight years ago had taken an assistant, contracting with him that in 1908 he would be admitted to the firm as a member upon certain stipulated conditions, among which was the payment of a considerable sum of money. Should he fail to fulfill the contract the assistant was not to be allowed to practice dentistry for the next two years within a radius of 100 English miles from Frankfort unless he paid to the firm 30,000 marks (about $7,000).

The assistant, after unsuccessful negotiations with the firm to permit him to practice on payment to it of a lesser sum, brought suit in the district court for the abrogation of the contract, claiming that such stipulations for preventing competition were against good morals and public utility. He rested his claim upon a decision rendered in a similar suit between authorized dentists by the supreme court of the empire, the highest legal tribunal in Germany, which had so declared.

The district court adopted this view and decided in favor of the plaintiff, holding that it is against the public interests when a dentist, in his feeling of responsibility, incumbent upon the exercise of his profession, is handicapped by stipulations which treat the professional activity from the point of view that it is solely a money making matter. Stipulations of this sort must therefore be considered as being injurious to the public good, and for this reason they should not be sustained.

This decision will give satisfaction to the public at large, as well as to aspiring American dentists. Germany and other European countries offer excellent chances of success to dental graduates. American dentists in good practice in the leading European cities have a larger professional income than their colleagues who practice in the United States.—Consular Report. in *American Journal.*

**Sodium Dioxid.** Some four years ago, in the *Items of Interest,* I published my observations on the effect of sodium dioxid as an obtundent of sensitive dentin. Since that time I have noted the reports of sundry observers whose experiences confirm my own—that it is without doubt valuable in such cases.

Its value, however, is not confined to the bleaching of teeth, disinfecting of canals and obtunding sensitive dentin. I find it most useful in those cases where a large cavity has become filled with hypertrophied tissue, which usually is hard to differentiate between gum and pulp tissue, and which ordinarily calls for pressure by gutta-percha or the lancet to remove. Either of these processes is attended with more or less pain and bleeding. By using the sodium dioxid with the care due an escharotic whose action is so hard to limit these growths may be removed speedily, almost painlessly, and without bleeding. My method is to dip a small piece of cotton into some of the powder on a slab, carry it to the affected tissue, which is protected by a cotton roll on each side, cover with a large piece of cotton and wait some few minutes, holding to place. Then remove, syringe with a large amount of water and repeat until the growth is gone. I have seen no bad results whatever following this method when used with the discrimination necessary while handling so powerful a drug. Another use is in those cases where the pulp has lost nearly all its vitality from severe inflammation but still is too sensitive to drill out and septic enough to cause danger if pressure anesthesia is used.

A small drill is passed into the pulp chamber if no opening exists, a particle of the powder put in and worked gently around the bottom of the hole thus made, and the pulp chamber can gradually be enlarged by burs, using the $Na^2$

$O^2$ when necessary with a minimum of pain and no danger of infecting the pulp and canals with the instruments used, and the drills will cut faster and cleaner.

Sodium dioxid is a most valuable agent if used as Dr. Soule suggests. There are many conditions in dental therapeutics where no other agent will answer the purpose so well. It should never be used, however, especially without the dam, unless the operator has vinegar or a weak solution of acetic acid at hand to neutralize its action on soft tissue. —E. M. SOULE in *Dental Digest*.

